# Pediatric Solid Cancers: Dissecting the Tumor Microenvironment to Improve the Results of Clinical Immunotherapy

**DOI:** 10.3390/ijms25063225

**Published:** 2024-03-12

**Authors:** Cristina Belgiovine, Kristiana Mebelli, Alessandro Raffaele, Marica De Cicco, Jessica Rotella, Paolo Pedrazzoli, Marco Zecca, Giovanna Riccipetitoni, Patrizia Comoli

**Affiliations:** 1 Dipartimento di Scienze Clinico-Chirurgiche, Diagnostiche e Pediatriche, University of Pavia, 27100 Pavia, Italy; 2 SC Chirurgia Pediatrica, Fondazione IRCCS Policlinico San Matteo, 27100 Pavia, Italy; 3 SSD Cell Factory e Center for Advanced Therapies, Department of Woman and Child Health, Fondazione IRCCS Policlinico San Matteo, 27100 Pavia, Italy; 4 SC Pediatric Hematology/Oncology, Department of Woman and Child Health, Fondazione IRCCS Policlinico San Matteo, 27100 Pavia, Italy; 5 Medical Oncology, Fondazione IRCCS Policlinico San Matteo, 27100 Pavia, Italy; 6 Department of Internal Medicine, University of Pavia, 27100 Pavia, Italy

**Keywords:** pediatric solid tumor, tumor microenvironment, immunotherapy, CAR-T cells

## Abstract

Despite advances in their diagnosis and treatment, pediatric cancers remain among the leading causes of death in childhood. The development of immunotherapies and other forms of targeted therapies has significantly changed the prognosis of some previously incurable cancers in the adult population. However, so far, the results in pediatric cohorts are disappointing, which is mainly due to differences in tumor biology, including extreme heterogeneity and a generally low tumor mutational burden. A central role in the limited efficacy of immunotherapeutic approaches is played by the peculiar characteristics of the tumor microenvironment (TME) in pediatric cancer, with the scarcity of tumor infiltration by T cells and the abundance of stromal cells endowed with lymphocyte suppressor and tumor-growth-promoting activity. Thus, progress in the treatment of pediatric solid tumors will likely be influenced by the ability to modify the TME while delivering novel, more effective therapeutic agents. In this review, we will describe the TME composition in pediatric solid tumors and illustrate recent advances in treatment for the modulation of immune cells belonging to the TME.

## 1. Introduction

The well-known role of T cells in the durable control of cancer has been impressively exploited in recent times through the therapeutic use of tumor-targeted advanced-therapy medicinal products (ATMPs), such as T cells that have been genetically modified to express chimeric antigen receptors (CARs) or natural T cell receptors (TCRs) bearing tumor-associated antigens [1]. Moreover, improved knowledge of cancer’s immune evasion mechanisms and host–tumor interactions [2] has led to the development of immune checkpoint inhibitors (ICIs), a class of agents that are able to counteract the suppressive effects of the tumor microenvironment (TME) on host immune cells. These compounds were able to achieve long-lasting remissions in previously unresponsive tumors and have rapidly become cornerstones of anticancer treatment [3].

The significant recent advances in the control of selected adult cancers [3] and adult and pediatric hematologic malignancies [4] have not been replicated so far in the setting of pediatric solid tumors. The causes consist, on the one hand, of the rarity of pediatric solid tumors, which, despite international efforts at cooperation, have resulted in major difficulties in developing novel therapies and conducting randomized clinical trials. In addition to this bottleneck, another major obstacle is represented by the peculiarities of pediatric tumors, which differ from adult cancer in terms of their mutational burden, underlying etiopathogenesis, and TME characteristics. Several studies have highlighted the critical role of the TME in response to therapy and the development of resistance in pediatric solid tumors [5]. Therefore, a better understanding of TME composition and function is crucial for the development of personalized and effective therapies in pediatric solid tumors. In this review, we will give an overview of the TME in the main pediatric solid cancers, highlighting how TME peculiarities may help personalize treatment for these tumors to optimize disease outcomes and illustrate the results of clinical trials that have attempted to modulate the TME using ICIs or ATMPs.

## 2. Overview of Solid Cancers in Childhood

The incidence of pediatric solid tumors in Europe varies depending on the type of cancer, patient age, and country [6]. Tumors of the brain and central nervous system are the most common pediatric solid tumors, followed by neuroblastoma, soft-tissue sarcoma, Wilms tumor, and bone tumors. Thanks to advances in diagnosis and treatment, survival rates have significantly improved in recent years. According to the National Cancer Institute, the overall 5-year survival rate for children with all types of pediatric solid tumors is about 75%. However, very rare childhood solid tumors are lacking in treatment options, with many of these diseases continuing to have a dismal prognosis.

The cellular origin of childhood solid tumors is diverse, as they can arise from different cell types, including embryonic or fetal cells [7], and their mutational burden is low [8], likely as a consequence of their manifesting in precursor cells of non-self-renewing tissues that have accumulated a lower number of mutations than that of cells of origin in adult tumors [9]. Thus, the genomic-based alterations and mechanisms in pediatric cancer are different from those observed in adult tumors, often being driven by the epigenetic deregulation of gene transcription, rather than the accumulation of somatic gene mutations [10], and showing unique genetic mutations that distinguish them from adult solid tumors [11]. Indeed, a significant proportion of pediatric brain tumors are driven by mutations in genes such as TP53, BRAF, and IDH1/2, which are less common in adult brain tumors. Similarly, structural changes, such as gene fusions and specific rearrangements, have been found to play a role in childhood cancer pathogenesis: EWSR1 in Ewing’s sarcoma, PAX3/7-FOXO in rhabdomyosarcoma, and MYCN or ALK in neuroblastoma [10]. Some of these genomic alterations have been associated with prognosis, allowing risk stratification and translation into therapeutic reduction or intensification, leading to a direct impact of genomic studies in pediatric cancer.

As knowledge on the molecular basis of tumorigenesis is being built, thanks to the efforts of international consortia, progress in drug development and early-phase clinical trials for targeted therapies in pediatric oncology is advancing. While some targeted therapeutic approaches specific to molecular alterations in pediatric cancer are being tested, other strategies for implementing treatment may rely on the modulation of the TME and the enhancement of protective cellular immunity. The success of ICIs largely depends on the presence of tumor-infiltrating lymphocytes that are specific to tumor-associated antigens and are able to mediate cancer cell lysis. As pediatric cancers have been described to have a low mutational burden, they generally have only a limited set of neoantigens that may be recognized and targeted by T cells, and the TME shows paucity in infiltrating T cells. Consequently, the activity of ICIs against any of the common solid cancers occurring in children and adolescents is suboptimal, and it is difficult to find suitable targets for the development of ATMPs. A way to overcome this situation could be to combine different strategies, such as ATMPs and ICIs, and/or act on other components of the TME to reduce its immune-suppressive and tumor-promoting effects.

## 3. The TME in Solid Tumors

**Figure 1:**
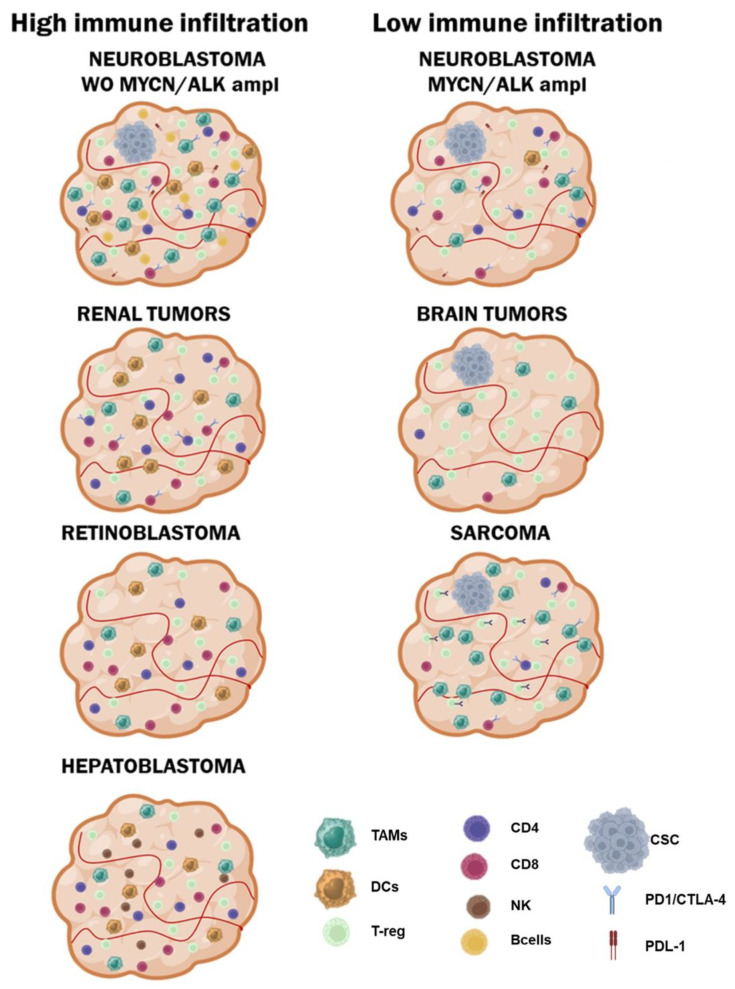
Characteristics of the TME in pediatric cancers. TAM: tumor-associated macrophage; DCs: dendritic cells; Treg: regulatory T cells; CD4: CD4+ tumor-infiltrating lymphocyte; CD8: CD8+ tumor-infiltrating lymphocyte; NK: natural killer cell; Bcells: B lymphocytes; PD-1: programmed cell death protein-1; CTLA-4: Cytotoxic T-lymphocyte antigen 4; PDL-1: Programmed death-ligand 1.

The tumor microenvironment is a complex ecosystem of cellular and non-cellular components that surround and interact with cancer cells. The composition of the TME is a critical determinant of tumor progression, metastasis, and response to therapy, and its study is central to amelioration of results in cancer (Figure 1). The immune TME is composed of different cell types that play a role in tumor proliferation and progression depending on their inherent functions and the cytokines or inhibitory ligands that they express. In pediatric solid tumors, the TME is highly heterogeneous, dynamic, and distinct from that of adult tumors.

### 3.1. The Non-Immune Component: Extracellular Matrix and Fibroblasts

The stromal part of the TME is composed of stromal cells, cancer-associated fibroblasts (CAFs), and an extracellular matrix (ECM). While angiogenesis and the proteins involved have been studied in many tumors, other factors that affect immune cells present within the tumor, such as the composition of the cellular matrix, the presence of CAFs, the cytokines that they release, and the composition of the patient’s bone marrow niche, have only been studied in a limited number of tumors. Microvascularization and angiogenesis have been investigated in numerous pediatric tumors, and the levels of angiogenic factors and tumor angiogenesis in situ have been found to correlate with metastatic disease and poor prognosis in neuroblastoma, osteosarcoma, and rhabdomyosarcoma [12,13,14,15,16]. The protein VEGF, which is known for its role in angiogenesis, has also been detected in several pediatric solid tumors, such as neuroblastoma, Wilms’ tumor, Ewing sarcoma, osteosarcoma, and rhabdomyosarcoma [15].

Furthermore, it has been found that the expression of VEGF can be regulated by Wnt/b-catenin activation in Ewing sarcoma cells [17]. In osteosarcoma, VEGF expression is associated with CXCR4 expression, which is found in 67% of osteosarcomas, and the co-expression of these two proteins is linked to decreased patient survival. A similar correlation has been observed in rhabdomyosarcoma patients [18].

In various tumor types, CAFs have been found to play a role in tumor progression and metastasis [2]. For example, CAFs expressing MMP-2 and MMP-9 have been identified in osteosarcoma [19,20], and their increased presence in the tumor area has been associated with MMP-2 and MMP-9 expression [21]. MMP-9 has been found to recruit bone-marrow-derived leukocytes in the tumor microenvironment [22]. These cells produce several soluble factors that help to recruit immune cells into the tumor. Transforming growth factor beta (TGF-β), fibroblast growth factor 2, platelet-derived growth factor, and epidermal growth factor (EGF) are secreted not only by stromal cells but also by cancer cells. CAFs physically support the tumor by secreting the ECM, and they can regulate the turnover of the ECM. Fibroblasts also secrete different soluble mediators, such as vascular endothelial growth factor (VEGF), interleukin (IL)-6, C-X-C motif chemokine ligand 12 (CXCL12), TGFβ, tumor necrosis factor (TNF)-α, IFN-γ, stromal cell-derived factor-1α, EGF, galectin-1, and the transcription factor NF-κB [23,24,25,26,27]. In osteosarcoma, the overexpression of a hyaluronidase called KIAA1199 has been identified as a prognostic factor due to its role in the development and maintenance of cancer metastasis [28]. Similarly, the high expression of fibronectin or αvβ3, either individually or in combination, has been associated with osteosarcoma [29].

Within the TME of a neuroblastoma, one finds nontumor cells called neural-crest-derived progenitors that give rise to the tumor stroma. Knowledge of this genetic signature correlates with the presence of CAFs and is useful for stratifying the most aggressive tumors (4M/neuroblastoma) to improve the therapeutic approach by targeting stroma-associated processes [30].

The major non-cellular component in the TME is the ECM, which is mainly composed of collagen. ECM is necessary to sustain the structure of the solid tumor and to store several soluble mediators, creating the typical immunosuppressive niche [31]. In Ewing sarcoma cells, as described by Hawkins et al. [17], Wnt/b-catenin activation is associated with enhanced ECM production and angiogenesis due to Wnt signaling and tumor/ECM crosstalk. In osteosarcoma, which has abundant ECM deposition, studies of patient samples have confirmed the prognostic usefulness of ECM-related markers [28,29,32]. The composition of the extracellular matrix (ECM) has also been studied in osteosarcoma. Mintz et al. [32] discovered that resistant tumors may have an increased ability to express osteoclastogenesis, tumor progression, and ECM remodeling genes. This suggests that chemotherapy-resistant osteosarcoma tumors mediate tumor growth and survival through altered proteolytic mechanisms that function in a multigenic fashion to activate osteoclasts, promote tumor survival, and modify the environment of the ECM.

Additionally, the inhibition of the collagen-crosslinking enzyme lysyl oxidase-like 2 reduces tumor growth and metastasis in preclinical models [33].

### 3.2. Cancer Stem Cells

Cancer stem cells (CSCs) are a subset of cancer cells that possess self-renewal and differentiation capabilities, contributing to tumor initiation, progression, metastasis, and resistance to therapy. In the TME of solid tumors, CSCs interact with various stromal cells, ECM components, and soluble factors, modulating the TME’s composition and function. CSCs can secrete cytokines, chemokines, growth factors, and ECM components, promoting angiogenesis, immune evasion, and ECM remodeling and creating a niche that facilitates their survival and expansion [34]. Furthermore, CSCs can communicate with immune cells, including T cells, natural killer (NK) cells, and myeloid-derived suppressor cells (MDSCs), leading to an immunosuppressive TME that impairs antitumor immune responses. A low concentration of CSCs is correlated with increased immunocyte infiltration [35]. Targeting CSCs and their interactions with the TME could provide new therapeutic approaches for solid tumors [36,37,38]. In pediatric solid tumors, CSCs have been identified in various types of cancers, including neuroblastoma, medulloblastoma, Ewing sarcoma, osteosarcoma, and rhabdomyosarcoma [39]. In neuroblastoma, the presence of CSCs modulates the TME in angiogenesis, in the formation of the ECM, in the presence of CAFs, and in immune cell interactions [40].

### 3.3. What Remains to Be Elucidated in the Study of the Non-Immune TME

In the study of pediatric tumors, the characterization of the stromal compartment and its contribution to pediatric tumor progression and metastasis is an understudied area that warrants further research. It is widely recognized that the crosstalk between tumor cells and stromal cells, such as fibroblasts and endothelial cells, contributes to tumor growth and progression. Increased expression/activity of the transcriptional co-regulator Yes-Associated Protein (YAP) following chemotherapy and relapse promotes resistance to therapy, including resistance to anti-disialoganglioside 2 (anti-GD2) immunotherapy, in high-risk neuroblastoma through the transcriptional repression of genes that play a role in the TME [41,42]. However, the role of YAP expression, as well as that of other factors in other solid tumors, has not been thoroughly evaluated.

The study of the hematopoietic niche of bone marrow cells has been primarily focused on patients with osteosarcoma due to the proximity of the tumor to the niche itself. It is known that the bone marrow microenvironment influences the dissemination of tumor cells from different origins [43,44]. Mesenchymal stem cells derived from the bone marrow have been shown to promote primary tumor growth and invasion [45], potentially inducing stemness and chemoresistance via the NFκB pathway and IL6 secretion [46]. Deepening the study of the hematopoietic bone marrow niche could be important in those tumors with secondary localizations or metastases to the bone marrow itself, such as in brain tumors [47], neuroblastoma [48,49], and sarcoma [47,50].

### 3.4. Immune Characteristics of the Tumor Microenvironment in Solid Tumors: Antitumor Properties

Tumor-infiltrating lymphocytes (TILs), which include CD4+ helper cells and CD8+ cytotoxic T-cells (CTLs), represent the adaptive immune response in the TME. CTLs have a potent antitumoral function, and their presence has been correlated with a better prognosis in several tumor settings [51]. Naive CD8+ T lymphocytes, which are primed and educated by professional antigen-presenting cells (APCs), can recognize tumor cells in an antigen-specific manner and mediate direct killing through the release of cytotoxic molecules, such as perforins or granzyme. Under physiological conditions, CD8+ activation occurs in secondary lymphoid organs, such as lymph nodes, but it can also occur in organized tertiary lymphoid structures within the tumor [52], where the antitumor immune response can be orchestrated [53]. CTLs are usually contained by a negative feedback loop once the cytotoxic function is completed. However, in cancer, there is persistent stimulation of CD8+ T cells in the TME, resulting in T cell exhaustion. There is a progressive loss of effector function, such as a decrease in IL-2, TNF-α, and IFN-γ production, and an increase in the expression of inhibitory receptors, such as PD-1, CTLA-4, Tim-3, LAG-3, B- and T-lymphocyte attenuator or BTLA [54]. For this reason, several therapeutic approaches that have been developed in the last twenty years are based on augmenting the natural immune response [55].

CD4+ T helper-1 cell population subsets may also contribute to tumor cell control through the induction of apoptosis and are responsible for sustaining the antitumor immune response of CD8+ T cells via the release of cytokines that are essential for T cell proliferation, as well as for macrophage recruitment and activation [56].

Tumor cells escape immune surveillance through the loss of class I major histocompatibility complex molecules on their surface. Class-I-associated antigen presentation is the cornerstone of CD8+ T cell effector functions; however, HLA class I downregulation promotes NK cell recognition and killing [57]. The NK subset belongs to the innate immune system and is highly efficient in identifying and killing undifferentiated or poorly differentiated tumor cells in both tumors and circulation [58,59]. NK cells can counteract the hostile TME [60,61,62] due to their ability to mediate cytotoxicity through both activating and inhibitory signals [63]. NK cells can kill tumor cells by releasing perforins and granzyme B, which induce necrotic or apoptotic cell death, and via the secretion of different antitumor cytokines, such as IL-10, IL-5, IL-13, granulocyte–macrophage colony-stimulating factor, IFN-γ, and TNF-α, which help to re-modulate the immunosuppressive environment present at the tumor site [64,65].

### 3.5. The Tumor Microenvironment in Solid Tumors: The Pro-Tumor Role

Within the immune landscape of the TME, cells belonging to the myeloid compartment appear to have a pro-tumor role. In general, both tumor-associated macrophages (TAMs) and tumor-associated neutrophils have a markedly immunosuppressive phenotypic profile. These cells, together with immature granulocytic and monocytic cells (myeloid-derived suppressor cells (MDSCs), favor tumor progression because they help orchestrate extracellular matrix remodeling, angiogenesis, tumor cell proliferation, metastasis, and immunosuppression, in addition to promoting resistance to chemotherapeutic agents and checkpoint blockade immunotherapy [66].

Macrophages (CD68+ cells) are specialized cells of the innate immune system that are characterized by their great plasticity. They are recruited within tumors via soluble factors produced by stromal and tumor cells, such as IL-3, colony-stimulating factor 1, and chemokine ligand-2 [67]. They are characterized by a high degree of plasticity and, as such, may be polarized towards more inflammatory functions, with antitumor activity (M1-like) or, conversely, anti-inflammatory activity with a pro-tumor role (M2-line; CD163+ or CD206+), although this distinction represents the extremes of a much broader spectrum of activation [68]. The cytokines that drive macrophages towards their inflammatory function the most are IL-2, IFN-γ, and TNF-α; however, other cytokines are present inside tumors, such as IL-4, IL-10, and IL-13, which lead macrophages to be more anti-inflammatory. In general, their role is related to the ability to impair CD8+ T cell infiltration, secrete IL-6, IL-8, and IL-10, and produce matrix metalloproteinases (MMPs). All of these events contribute to the creation of an immunosuppressive microenvironment that supports tumor growth and metastasis [69]. High infiltration of TAMs within the TME is associated with a poor prognosis in many tumor types, and their targeting or remodeling is considered a promising strategy [66].

Neutrophils also have a role in tumors; their phenotype depends on the chemokine/cytokine composition of the TME, and they may have both antitumor (N1) and pro-tumor (N2) phenotypes. Their antitumor functions include the production of reactive oxygen species, but in the later stages of tumor development, there is high infiltration of N2 neutrophils that support tumor growth and progression via the degradation of arginine, an activator of T cells, or suppress IL-18 production [70].

The cells assigned to prime CD8 T cells are mainly tumor-infiltrating dendritic cells (DCs) that can scan and phagocytize tumor cells, process tumor-associated antigens, and present antigen-derived peptides within HLA class II molecules. Although their function is usually associated with tumor inhibition, the presence of some cytokines drives them toward a pro-tumor role. The presence of IL-6, IL-10, IDO, macrophage colony-stimulating factor, TGF-β1, prostaglandin E2, and VEGF can impair the ability of DC to present antigens and give them an immunosuppressive phenotype [71]. DC-based therapeutic strategies strive to inhibit pro-tumor cytokines or are directed to the creation of personalized vaccines [72].

The only lymphoid subset that can promote tumor progression is the regulatory CD4+ T lymphocyte subset (Treg), which directly secretes or facilitates the formation of immunosuppressive molecules (e.g., IL-10, adenosine) and modulates the APC function (e.g., via CTLA-4–CD80/86 interactions) [73].

## 4. TME Immune Profiling in Pediatric Solid Tumors

**Figure 2:**
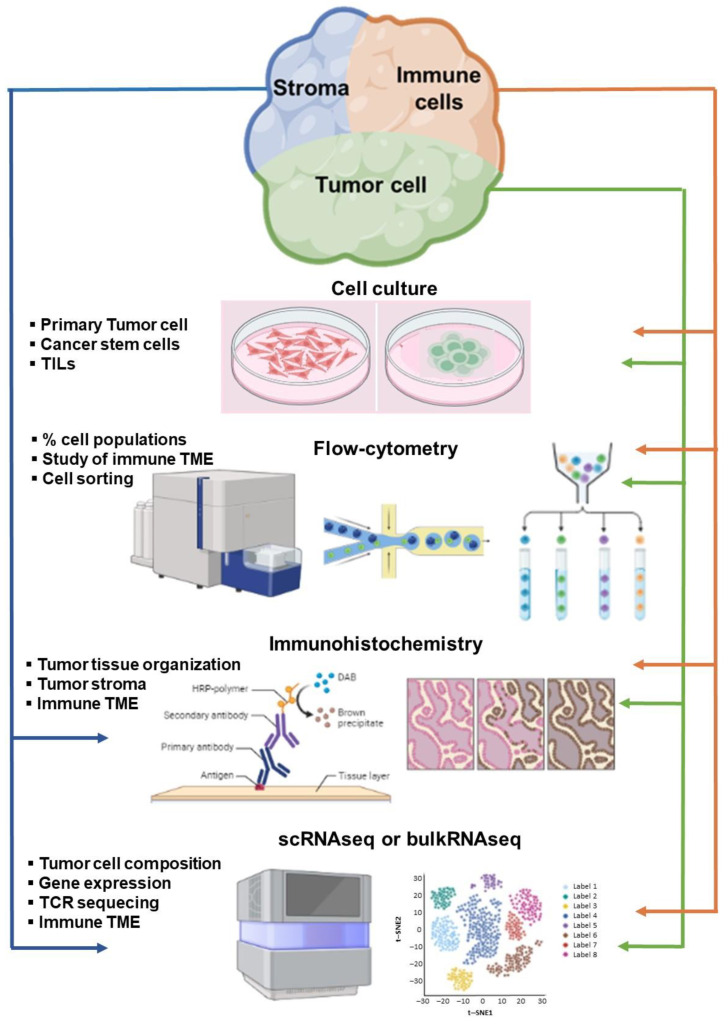
Tools for studying the TME in cancer. TILs: tumor-infiltrating lymphocytes; TME: tumor microenvironment; scRNA seq: single-cell RNA sequencing; bulkRNA seq: bulk cell population RNA sequencing.

Tumor immune profiling has proven useful in adult cancer to predict responses to immunotherapy, at least in some tumor types. However, data obtained from adults are not always transferable to the pediatric setting, as childhood cancers are biologically different. A high tumor mutation burden, a predictor of response in adults, is uncommon in pediatric tumors [74]. On the other hand, peculiar genomic features driving neoantigen generation and the high thymic output observed in children, with the potential for the emergence of new T cells, warrant specific studies in pediatric cohorts (Figure 2). So far, a limited number of immune profile studies have been performed on childhood solid tumors to guide stratification and predict responses to immunotherapy (as summarized in Table 1).

### 4.1. Brain Tumors

Glioma and medulloblastoma are the most common types of solid tumors in children [75]. Gliomas are a group of primary brain tumors of glial tissues that include astrocytoma, oligodendroglioma, and glioblastoma. Among these, glioblastoma is the most commonly occurring malignant primary brain carcinoma [76]. Analysis of immune cell infiltration in pediatric gliomas demonstrated that myeloid cells are major components of the TME and show enrichment of CD8+ T cells and CD45+ leukocytes in low-grade gliomas compared to high-grade gliomas [77]. While the percentage of tumor-associated CD68+ macrophages is comparable across subgroups, diffuse midline gliomas have the lowest number of infiltrating CD8+ T cells and CD163+ macrophages, which contributes to their immunosuppressive phenotype [78].

Medulloblastoma accounts for 15% to 20% of pediatric CNS neoplasms. Therapy for medulloblastoma has evolved into well-accepted multimodal regimens that include maximal surgical resection, craniospinal irradiation (CSI), and polychemotherapy based on the use of etoposide, methotrexate, platin, and thiotepa, depending on the risk stratification, with curing being observed in up to 70% to 80% of patients according to the subtype [79]. A medulloblastoma subgroup displayed activation of sonic hedgehog signaling, which is implicated in suppressing the immune system and promoting an immunosuppressive tumor microenvironment. The sonic hedgehog subgroup was the most enriched in CD163+ macrophages, suggesting differential roles of TAMs in this pathological entity.

Comparing immune cell infiltration in the peritumoral area and tumor core of glioblastomas showed that CD163+ cells were more abundant in the tumor core, similarly to the higher expression of the immunosuppressive markers PD-L1, IDO, and TIGIT [80].

**Table 1:** Studies on the immunoprofiles of pediatric solid tumors.

Tumor Type	Technique	Findings	Sample n.	Ref.
Brain tumors	FC	Enrichment of CD8+ T cells and CD45+ leukocytes in low-grade gliomas compared to high-grade gliomas. Lowest number of infiltrating CD8+ T cells and CD163+ macrophages in diffuse midline gliomas.	62	[77,78]
	FC; CA	Low macrophage or T-cell infiltration and PD-1.	59	[81]
	FC	Higher immune cell infiltration, more immunosuppressive (HLA-DR+ and CD64+) in pilocytic astrocytoma and ependymoma than in glioblastoma and medulloblastoma.	37	[82]
	IHC and Microarray	Low-level PD-L1 in medulloblastoma patients.	44	[83]
	rtPCR, IHC, FACS, WB	PD-L1 expressed in both a tumor and myeloid in a subgroup of ependymoma patients; PD-1 levels found in both CD4 and CD8 T cells	76	[84]
	FACS, rtPCR	High rate of infiltrating Tregs expressing CTLA-4	10	[85]
	IHC	PD-L1 expression in glioblastoma samples (36%)	14	[86]
NBL	WTS	High TIL infiltrate markers and higher expression of PD-1 in MYCN-A tumors	657	[87]
	RNAseq (Systematic analysis)	High T cells, CD8+ T cells, T17, NKT cells, T1 cells, Treg cells, and DCs in high-risk neuroblastomas without MYCN-A	408	[88]
	RNAseq	High levels of macrophages, B cells, and inflammation genes in neuroblastomas without MYCN-A	129	[89]
	IHC	PD-L1 expression in neuroblastoma samples (14%): associated with inferior survival and a higher number of macrophages	118	[86]
	FACS	PD-1 on αβ and γδ T lymphocytes and NK cells in metastasis samples	19	[90]
	IHC	Low–moderate levels of PD-1 and PDL-1	31	[91]
Sarcomas	IHC	Higher percentage of granulocytes and fewer lymphocytes. Increased expression of CTLA-4, CD14+HLA-DR^lo/neg^, and TNFRII on CD14+.	19	[92]
	IHC	PD-L1 expression found in epithelioid sarcoma (100%), synovial sarcoma (53%), rhabdomyosarcoma (38%), and Ewing sarcoma (33%); correlated with worse overall survival	82	[93]
	IHC	Low–moderate level of PD-1 and absence of PDL-1	68	[91]
	CIBER SORT	43% macrophages, mainly M2 type 23% T cells	197	[94]
	FACS, IHC	Elevated CD14+ HLA-DRlo/neg cells, elevated CTLA-4+ T cells, and decreased CD4 T cells	74	[92]
	rtPCR, FACS, IHC	Higher expression of CTLA-4 in circulating CD4 and CD8 T lymphocytes	6	[95]
Renal tumors	IHC	Low–moderate level of PD-1 and absence of PDL-1	25	[91]

FC = flow cytometry; CA = cytotoxic assay; WTS = whole transcriptome sequencing; NBL: neuroblastoma; MYCN-A: MYCN-amplified; IHC: Immuno-histochemistry.

Ependymomas are primary tumors of the central nervous system that arise along the ventricular system and spinal cord. In pediatric patients, ependymomas comprise ~10% of primary central nervous system tumors, with the majority arising in the posterior fossa [96]. Pediatric ependymomas with higher infiltration of CD3+ and CD8+ T cells in the microenvironment at diagnosis had a longer progression-free survival, while elevated Forkhead box P3 regulatory T cells and CD68+ macrophages were correlated with a shorter survival rate [78].

Griesinger et al. showed differences in immune infiltration and the degree of immune suppression in biopsy samples from patients diagnosed with pilocytic astrocytoma, ependymoma, glioblastoma, and medulloblastoma [82]. Compared with glioblastoma and medulloblastoma, pilocytic astrocytomas and ependymomas had significant myeloid (characterized as CD45+CD11b+) and lymphocyte infiltration. Low levels of PD-1 were exhibited by the infiltrating immune cells, which suggested a more permissive TME for immunotherapy. Overall, most brain tumors are cold from an immunological point of view, with high myeloid signatures and low T cell infiltration, as well as with particularly aggressive forms such as diffuse intrinsic pontine glioma and medulloblastoma. However, there is evidence that some subtypes are more inflamed than others and may respond to immune checkpoint inhibitors [97]. It will be critical to perform further studies on brain tumors to understand the complexities of the immune microenvironment more deeply and to provide therapeutic decisions and better outcomes.

### 4.2. Neuroblastoma

Neuroblastoma is a neural crest-derived malignancy of the peripheral nervous system and is the most diagnosed extracranial solid tumor in infancy. It is characterized by clinical heterogeneity with a disease spectrum ranging from spontaneous regression without any medical intervention to treatment-resistant tumors with metastatic spread and poor patient survival [98]. Neuroblastoma patients are stratified into low-, intermediate-, and high-risk groups based on different parameters, including tumor histology, clinical stage, tumor cell ploidy, and MYCN oncogene amplification, which are present in 20–25% of the cases and are correlated with high-risk disease and poor prognosis [99]. Multimodal treatment is modulated according to risk stratification and is based on the use of surgical excision, chemotherapy, radiotherapy, hematopoietic stem cell transplantation, oral retinoic acid, and immunotherapy with anti-GD2 monoclonal antibodies, the latter being used both up front and in relapse.

MYCN amplification and ALK mutations promote tumor angiogenesis and vasculogenesis through the secretion of vascular endothelial cell growth factor (VEGF) by cancer cells and other cells residing in the TME, such as mesenchymal stromal cells (MSCs) and endothelial cells. In addition to these factors, the TME substantially contributes to the biology and outcome of neuroblastoma. It has been shown that patients with non-MYCN-amplified metastatic neuroblastomas had higher infiltration of TAMs than locoregional tumors did [100]. Moreover, infiltration with Th2-driven macrophages expressing CD163 and CD206 was also observed in a subset of high-risk neuroblastoma tumors with the deletion of chromosome 11q [100]. High telomerase activity and, consequently, replicative immortality could be controlled by the TME and, in particular, by inflammatory monocytes/macrophages through miR release, while the activation of STAT3 by IL-6 and sIL-6R produced by MSCs and TAMs induced the expression of several pro-survival proteins such as survivin, MCL-1, and Bcl-XL, which caused resistance to chemotherapeutic agents [101].

Several studies on immune profiling in neuroblastoma while also considering its risk classification have been published. A recent comprehensive study of deep RNA-seq for pretreatment diagnostic tumors from 129 high-risk and 21 low- or intermediate-risk patients revealed the complex microenvironment that may exist in high-risk neuroblastoma, and the patients could be layered into three groups based on their immune profiles. High-risk tumors that were not MYCN-amplified (clusters 3 and 4) had higher T-cell signatures and TCR heterogeneity and increased expression of immune checkpoints in comparison with those of high-risk MYCN-amplified tumors (cluster 1) [89]. Brohl et al. performed an immunogenomic analysis on a cohort of 657 tumor samples from 623 pediatric or young adult patients diagnosed with an extra-cranial solid malignancy, representing 14 diagnoses. They noticed that intra-tumoral clonal T-cell infiltration was correlated with patient survival in the cases of neuroblastoma and osteosarcoma [87]. From a systematic analysis of public RNA-seq data (TARGET) on the TME composition in neuroblastoma, it was found that CD8+ T cells, T-helper 17 cells, NKT cells, Tregs, and DCs were significantly more enriched in the group with high-risk neuroblastoma without MYCN amplification compared to the group with high-risk neuroblastoma with MYCN amplification (*p* < 0.05). Moreover, T follicular helper cells (TFHs) showed a significant positive association with survival in high-risk neuroblastoma without MYCN amplification [88].

CTLA4 (cytotoxic T-lymphocyte-associated protein 4) is a protein that plays a critical role in regulating immune responses. It is primarily expressed in T cells but can also be found in other immune cells. In neuroblastoma, CTLA4 expression has been detected in both tumor cells and immune cells within the tumor microenvironment. A study by Kushner et al. [102] demonstrated that neuroblastoma tumor cells expressed CTLA4, and higher levels of CTLA4 expression were associated with poorer patient outcomes. The study also showed that infiltrating immune cells in the tumor microenvironment, such as T cells and macrophages, expressed CTLA4, suggesting a potential role for CTLA4 in regulating the immune response to neuroblastoma.

With a better knowledge of the contribution of the TME to the progression of neuroblastoma and of its mechanisms, clinical trials testing TME-directed agents have been initiated; these have targeted TME cells that contribute to a pro-tumorigenic environment, signaling pathways of crosstalk between tumor cells and the TME, or cancer cells through immunotherapy.

Moreover, the study of the TME’s immunosuppressive features in this type of tumor has prompted research groups to explore techniques such as repolarization through mesenchymal stromal cell (MSC) delivery systems [103]. By studying immune populations, researchers have also been able to identify the resistance mechanism of Celyvir viral oncolytic therapy, which is mediated by T-cell exhaustion induced by intratumoral myeloid cells [104].

### 4.3. Sarcoma Family Tumors

Sarcomas are a heterogeneous group of tumors that can arise in bone or soft tissues around the body. Together, they account for about 10% of pediatric cancers [105]. Osteosarcoma, Ewing sarcoma, and rhabdomyosarcoma are the most common pediatric sarcomas.

Osteosarcoma is a high-grade primary skeletal malignancy characterized by spindle cells of mesenchymal origin depositing an immature osteoid matrix. With an annual incidence rate of 3.1 cases per million in the US, osteosarcoma accounts for less than 1% of all newly diagnosed cancers in adults and 3–5% of those in children, but it is the most common primary malignancy in adolescents besides leukemia and lymphoma [106]. Alveolar rhabdomyosarcoma and embryonal rhabdomyosarcoma are the most common soft tissue sarcomas, accounting for about 5% of childhood cancers [107]. Ewing’s sarcoma represents a rare, highly malignant disease, with most patients harboring a priori micro metastases, since, without systemic therapy, over 90% of patients die from disseminated disease [108].

Compared with other cancer types, osteosarcoma exhibits an immune-active phenotype characterized by increased mean enrichment of transcripts for immune cell infiltration [106]. Enrichment scores for some leukocyte populations, such as TFHs, DCs, neutrophils, macrophages, and monocytes, were associated with significantly improved prognosis, while B cell and gamma delta T-cell enrichment was associated with significantly worse survival in this cancer type [88]. Conversely, the most abundant cells in the TME of Ewing’s sarcoma were immunosuppressive M2-type macrophages, and increased numbers predicted a shorter event-free survival (EFS) [94]. Hingorani et al. studied the immune population present in the bloodstream of pediatric osteosarcoma and Ewing sarcoma patients and found an increased expression of CTLA-4 in both CD4+ and CD8+ T cells, together with a population of CD14(+) HLA-DR(lo/neg) immunosuppressive monocytes, which were correlated with advanced disease [92]. Looking at tumor tissue, they found massive infiltration of CD14+ monocytes in osteosarcoma compared to Ewing sarcoma but found limited T cell infiltration [92]. A study by Contardi et al. found that CTLA4 was also expressed on osteosarcoma tumor cells from six primary specimens that were examined [109], although the role of this molecule in tumor cells remains to be ascertained [109]. MSCs have been utilized in osteosarcoma to carry an oncolytic virus (O-Ad) along with G-CSF, leading to increased intratumoral TILs (tumor-infiltrating lymphocytes) and subsequent tumor reduction in in vivo experiments with mice [110].

### 4.4. Renal Tumors

Renal tumors in children include nephroblastoma (or Wilms tumor), which originates from nephrogenic progenitor cells from embryonic kidneys, and other rarer cancer types. The outcome for Wilms tumor is excellent for the majority of patients, but aggressive subtypes are challenging to treat and have a poorer prognosis, with a survival rate of approximately 50% or lower [111]. Data from the literature suggest that Wilms tumor is an immune-engaged tumor. An immune infiltrate analysis showed that CD8 and CD4 T cells were localized in a tumor and exhibited an activated phenotype [112], with the presence of activated CD8 T cells correlating with positive outcomes [113]. However, the TME may include immunosuppressive cells such as Tregs and increased plasmacytoid DCs. A high density of M2 macrophages within the TME was correlated with shorter overall survival and unfavorable histology [114], and the presence of immunosuppressive cytokines—in particular, TGFβ expression—was correlated with invasion/metastasis and disease progression [115]. Regarding other renal tumors, conventional and unconventional T cells were observed to infiltrate pediatric papillary renal cell carcinoma (PRCC) tumors and express CD1d, which presents potential therapeutic avenues [116].

Upregulation of PD-L1 is observed in 14–29% of Wilms tumors [117,118], and it is correlated with poor prognosis in aggressive subtypes, with a higher risk of recurrence in the case of favorable histology, independent of tumor stage [119]. Although a limited number of cases have been described, 50% of rhabdoid tumors show membranous expression of PD-L1 in 10–70% of tumor cells, with over 50% displaying high levels (>2/HPF) of tumor-infiltrating lymphocytes expressing PD-1 at levels ranging from 10 to 60%, which correlated significantly with tumor PD-L1 staining [120].

### 4.5. Retinoblastoma

Retinoblastoma is a rare intraocular malignancy representing approximately 3–4% of all pediatric malignancies [121]. Retinoblastoma typically develops in children under 5 years of age, and early diagnosis and prompt treatment can lead to a high cure rate. Late diagnosis, advanced clinical stage, and late age at presentation are the main risk factors for tumor-related mortality [122,123]. Enucleation is inevitable for tumors in the advanced group, despite the use of improved therapies, such as neoadjuvant chemotherapy [124].

The TME of retinoblastoma contains a range of immune cells, such as dendritic cells, monocytes, macrophages, and T-lymphocytes [125]. Studies have shown that decreased retinoblastoma cell proliferation is linked to increased immune cell infiltration [125]. Mao et al. identified two immunological subgroups of retinoblastoma based on the immune profiles of 28 types of immune cells. These subgroups of patients had different clinical characteristics and gene expression profiles [126]. By studying the distinct DNA methylation patterns between the two subgroups, Mao et al. found that immune cell infiltration was related to retinoblastoma migration and metastatic progression [126]. An integrative single-cell transcriptome and whole-exome sequencing analysis of retinoblastoma patients revealed that the TME in retinoblastoma was composed of tumor-associated macrophages (TAMs), astrocyte-like cells, and cancer-associated fibroblasts [127]. These TAMs created an immunosuppressive environment and could regulate tumor cells through specific signaling pathways. Chemotherapy modified the retinoblastoma microenvironment, as it was found to reorient the TME from an anergic state to an active, CD8+, PD-L1+ hot state [128].

### 4.6. Hepatoblastoma

Hepatoblastoma is a pediatric liver cancer that mainly occurs in the first three years after birth. It is characterized by β-catenin and Yap1 activation and overexpression, which may be involved in its pathogenesis. Despite an overall survival rate of up to 80%, hepatoblastoma remains a challenging disease, particularly in patients with advanced disease or poor prognostic factors. Recent studies have shed light on the immune modulation of hepatoblastoma patients [129]. Guo et al. found that children with hepatoblastoma had more NK cells and highly expressed killer cell immunoglobulin-like receptors, a protein that inhibits the cytotoxicity of NK cells, resulting in the immune escape of tumors [130]. Taurine Up-Regulated 1, an onco-lncRNA, mediates the infiltration of pro-tumor immunocytes in hepatoblastoma patients carrying the CTNNB1 mutation [131]. Methylation and epigenetic changes also influence the immune microenvironment in hepatoblastoma [132].

## 5. The Role of Immunomodulating Agents on TME

The immune response to cancer within the TME is regulated by a network of cells that secrete activating and inhibitory molecules. The final response derives from the net interactions within the network and can be modulated through therapeutic agents.

A crucial role is played by immune checkpoints (ICs), which are trans-membrane proteins expressed by immune cells that regulate the extent of a response. Among the ICs, those that have been more extensively studied are cytotoxic T-lymphocyte-associated antigen-4 (CTLA-4) and programmed cell death receptor 1 (PD-1), as well as its ligands PD-L1/2, but other molecules have also been investigated for their potential clinical use, such as lymphocyte-activating antigen-3 (LAG-3), T cell immunoglobulin and mucin-domain containing-3 (TIM-3), T cell immune receptor with Ig and ITIM domains (TIGIT), B7-H3, and indolamine dioxygenase (IDO) [54]. The development of ICIs represents an important milestone in the therapeutic control of cancer due to their ability to potentiate immune responses against tumor cells. In addition to ICIs, a few targeted therapies, including specific monoclonal antibodies directed to pediatric-tumor-associated antigens, have also been developed. One example is the chimeric monoclonal anti-GD2 antibodies dinutuximab and dinutuximab beta, which are considered to be the standard of care in the first-line treatment of children with high-risk neuroblastoma after induction chemotherapy and autologous hematopoietic stem cell transplantation, and they are used also to treat relapsed or refractory neuroblastoma patients with or without minimal residual disease positivity [133]. GD2 has restricted expression in normal tissues but is overexpressed across a wide range of tumors and has been discussed as a target in rare and aggressive pediatric malignancies such as Ewing’s sarcoma, osteosarcoma, and H3K27M-mutant diffuse midline glioma. However, anti-GD2 MoAb use in pediatric cancers other than neuroblastoma has been, so far, very limited [134].

The modulation of ICs is effective only in the presence of immune effector cells in the TME, but pediatric solid tumors often show a paucity of T cell infiltrate. For this reason, in addition to ICIs, adoptive cell therapy with tumor-directed natural T cells or gene-modified tumor-targeted cellular products may have an important role in cancer control. These novel therapeutics, which were proven effective in the control of hematologic cancer, are also being tested in the setting of pediatric solid tumors [135].

### 5.1. Immune Checkpoint Inhibitors in Pediatric Solid Tumors

ICIs reverse the immune effector cell inhibition mediated by tumor cells and the TME so that an immune response can be mounted against cancer [136]. In adult patients, a better response to ICIs correlates with the presence of effector T cells and higher tumor mutational burden (TMB) [137]. However, pediatric tumors generally present very low TMB due to the presence of few driver mutations, as well as their low immunogenicity and antigenicity [138]. Moreover, pediatric tumors have low TIL numbers and limited expression of PD-1, PD-L1, and PD-L2 [91]. TIL function and infiltration are impaired by the immunosuppressive environment created by MDSCs, tumor-associated macrophages, tumor-associated fibroblasts, and Tregs [139].

CTLA-4, the first immune checkpoint receptor to be clinically targeted, is expressed on the surface of active T cells and provides an inhibitory signal for T cells [136,140,141]. The interaction of the T cell receptor (TCR)/CD3 complex, the CD28 co-stimulatory signal, and the co-inhibitory signal CTLA-4 is necessary for normal T cell activation and subsequent effector control. To dampen T cell activation, CTLA-4 outcompetes CD28 for the shared B7 molecule ligands through a higher binding affinity. In cancer cells, the expression of CTLA4 is induced to evade T cell antitumor activity, and its blockade potentiates active immune responses against tumor cells and decreases immunosuppression by Tregs [140,141]. In pediatric tumors, the expression of CTLA-4 was detected in glioblastoma, neuroblastoma, and sarcomas, particularly in osteosarcoma, suggesting its potential role as a target for immunotherapy [92,95,102,109]. Preclinical studies have shown that treatment with CTLA4 in combination with other therapies, such as radiotherapy and targeted therapies, such as those with anti-GD2 MoAbs or with anti-PD-1, yields better results in terms of overall survival and reduction of tumor growth in a human model of neuroblastoma [142] and glioblastoma [143], as well as in an orthotopic model of glioblastoma in which the greatest curative effect was obtained using a combination of anti-CTLA-4 and anti-PD-1 antibodies in addition to oncolytic viruses [144]. This success was due to macrophage polarization and the increased ratio between infiltrating effector T cells and Tregs [144]. Two humanized anti-CTLA-4 antibodies, Ipilimumab and Tremelimumab, have been approved as therapeutic options for the treatment of cancer [145,146]. In a pediatric setting, Ipilimumab treatment was studied in a phase I trial in 33 patients under the age of 21 who had advanced solid tumors, such as melanoma, bladder, and renal cancer, neuroblastoma, and sarcoma (Table 2). The spectrum of immune-related adverse events was similar to that described in adults, although it was frequently noticeable after a single dose. Despite the absence of objective tumor regression, stabilization of disease was registered in four patients, and a higher overall survival rate was observed in subjects with immune-related toxicities [147].

A different immune checkpoint pathway includes PD-1 and its ligands PD-L1 and PD-L2. PD-1 is a protein that belongs to the CD28 superfamily and is a key regulator of programmed cell death in lymphocytes, so it has a critical role in maintaining peripheral tolerance [153]. Upon activation, PD-1 is expressed in different T cell subsets, B lymphocytes, NK cells, some myeloid cells, and cancer cells [153]. In the TME, PD-1 is also highly expressed in Tregs, increasing their activity and proliferation. PD-L1 and PD-L2, which are ligands of PD-1, are members of the B7 family; while PD-L1 is found in different lymphoid organ resident cells and many non-hematopoietic tissues, PD-L2 expression is limited to APCs [154]. The interaction of PD1 with PD-L1 hampers T cell activation through the suppression of T cell proliferation, survival, and cytokine secretion, causing the inhibition of antitumor lymphocytes that are present in the TME [154]. Most tumor PD-1/PD-L1 expression studies were performed on adult specimens, and few studies were directly performed on pediatric solid tumor biopsies. The observed expression of PD-1 and PD-L1 was variable according to different reports. Expression of PD-L1 was observed in alveolar rhabdomyosarcoma (86%), rhabdomyosarcoma (2–50%), neuroblastoma (14–72%), Ewing sarcoma (0–57%), osteosarcoma (5–47%), and glioblastoma multiforme (36%) [155]. A higher expression of PD-L1 was correlated with a worse prognosis [84,86,93].

The use of the anti-PD-1 antibodies Pembrolizumab and Nivolumab and the anti-PD-L1 antibodies atezolizumab, avelumab, and durvalumab has so far been mostly limited to adult solid tumors [156,157]. Despite encouraging safety and efficacy in pediatric hematological cancer, the results in pediatric solid cancer are, so far, disappointing. A phase I/II study of nivolumab as a single agent in children and young adults with lymphomas or R/R ST showed no objective responses in ST, with 33% and 50% disease stabilization in sarcomas and neuroblastoma, respectively, and grade 3–4 toxicities at a rate of 36% [150]. In another phase I/II study that is still ongoing (NCT02992964), two partial responses of a long duration were observed in pediatric patients with hypermutated ST [152]. A phase I/II trial of pembrolizumab described eight partial remissions in R/R ST, with grade 3–5 toxicities at a rate of 8% [149]. Regarding anti-PD-L1 agents, a phase I/II study of atezolizumab in pediatric patients and young adults with refractory or relapsed solid tumors showed partial remission in 1/75 patients treated for ST, with a good toxicity profile [148]. Likewise, a phase I study of avelumab described four stabilizations of disease in 21 patients treated for R/R ST, without grade 4–5 adverse events [158].

As the use of single agents has not been encouraging in pediatric ST, the prevention of immune escape through dual checkpoint blockage has been attempted. In an implanted mouse model of metastatic osteosarcoma, treatment with anti-PD-L1 antibody reduced the expression of PD-L1, increased the expression of CD80/CD86 in tumor cells, and increased the expression of CTLA-4 in tumor-infiltrating CD8+ T cells. Additionally, 50% of treated mice experienced complete protection from metastasis and T cell memory protection against future tumor inoculation following PD-1/CTLA-4 signaling inhibition with combined therapy [159]. As observed with anti-CTLA4 and anti-PD-1, better results were obtained in animal models when more than one immune checkpoint was targeted [160,161,162] or when other immune activation mediators, such as agonists of Toll-like receptor 3 (TLR3), TGFbeta, or standard radiation or chemotherapy, were associated [163,164,165].

Combinations of CTLA-4 and PD-1/PD-L1 pathway blockages are actively being investigated in pediatric patients [166]. A phase II study conducted in 55 pediatric and young adult patients with R/R ST showed four partial remissions in the young adult cohort and four disease stabilizations in children, with 11% showing dose-limiting toxicities, demonstrating the good tolerability of ICI combinations [151]. However, a phase I/II randomized trial comparing nivolumab alone with nivolumab + ipilimumab in pediatric patients with R/R solid tumors of the central nervous system failed to demonstrate an advantage for the ICI association [167]. Further trials are underway to test the feasibility and efficacy of ICI combinations in association with standard chemotherapy, radiotherapy, or other immune-modulating agents (Table 2).

### 5.2. ATMPs in Pediatric Solid Tumors

Several ATMPs are under investigation in clinical trials against solid tumors, but only a few have involved pediatric patients [168,169]. Most of these therapies are based on the use of T cells, but NK cells and monocytes are gaining increasing attention [170,171]. For purposes of this review, we shall focus our attention on T cell products and just briefly mention NK cells and monocyte ATMPs. T cell therapies for solid tumors can be classified into two broad categories: somatic cell therapies, with tumor-infiltrating lymphocytes (TILs) as the main subtype, and gene therapies, which are mainly obtained through the transfer of antigenic specificity through chimeric antigen receptors (CARs) or natural T cell receptors (TCRs) isolated from high-avidity T cells that recognize cancer antigens [169]. Somatic cell therapy with TILs has shown promising results in solid tumors [172]. However, at present, clinical trials are ongoing for adult solid tumor patients but not in the pediatric setting, which is probably due to the scarce presence of TILs within the pediatric TME. Somatic cell therapy with virus-specific T cells was employed in patients with virus-associated hematologic and solid tumors, such as Epstein–Barr-virus-related nasopharyngeal carcinoma or human-papillomavirus-related carcinomas [169,170,171,172,173]. 

TCR therapy trials have mostly targeted overexpressed self/tumor antigens that are also expressed by healthy adult cells, such as gp100 and Melan-A/MART-1, or oncofetal antigens that are present on healthy cells exclusively during fetal development and are ectopically expressed in tumors, such as melanoma antigen E or NY-ESO-1 [169]. Early-phase clinical trials showed some good partial responses, but dose-limiting on-target, off-tumor toxicity was an important limitation [174,175,176,177]. NY-ESO-1 expression was also studied in different pediatric tumors together with other cancer–testis antigens [178], but no clinical trials are presently under investigation for pediatric patients.

The use of CAR-T cells for the treatment of pediatric cancer patients has been steadily increasing. Despite the success stories in the control of pediatric hematological cancers, the efficacy of CAR-T in the setting of solid tumors has been hampered by the complex TME network, which may favor immune escape and induce therapeutic resistance [179].

CAR-T studies focusing on malignant solid tumors are limited in number in comparison to those on hematological malignancies, and very few have targeted the pediatric population (Table 3).

The first pediatric solid tumor targeted with CAR-T cells was neuroblastoma. N cells, as well as glioma, melanoma, and sarcoma cells, uniformly express the ganglioside GD2, which is the target of monoclonal-antibody-based therapeutic interventions [133]. In recent years, genetically engineered T cells modified with CARs specific to GD2 antigen have been produced and evaluated in clinical trials [181,183,184,185,186,187]. Autologous virus-specific T cells that expressed anti-GD2 CAR were used to treat neuroblastoma patients in an early clinical trial. Despite the use of a first-generation CAR construct and CAR-T cell infusion without lymphodepletion, objective responses were observed, with three patients reaching CR without dose-limiting toxicities [180,181]. A subsequent trial in the same group using a third-generation GD2–CAR and modified T cell administration in the absence or presence of lymphodepletion did not show an improvement in terms of efficacy, as the treatment proved safe, but disease stabilization was observed in only four patients after CAR-T therapy [183]. Similar results were obtained in a phase I trial that treated 12 patients with second-generation GD2–CAR-T cells administered with lymphodepletion [184]. Recently, a phase I/II study conducted in 27 patients with R/R neuroblastoma that employed third-generation GD2–CAR-T cells and lymphodepleting chemotherapy described very encouraging results, with more than 60% showing objective responses and an event-free survival of 36% at 3 years in the absence of DLTs [185]. An ongoing trial in R/R neuroblastoma patients with GD2–CAR-NKT cells co-expressing IL-15 administered after lymphodepletion showed safety and good efficacy, as in one of the first three patients treated with dose level 1, a PR was observed, with no DLTs [186]. GD2–CAR-T cells are also being used to treat children and adults with GD2-positive gliomas [187] and osteosarcoma (NCT03721068), with some clinical benefit.

Human epidermal growth factor receptor 2 (HER2)/Neu antigen-directed CAR-T cells are being tested in the settings of glioma, osteosarcoma, and nephroblastoma. Even in the presence of low-level HER2 expression, HER2–CAR T cells effectively identify and destroy cancer cells [188] and have proved effective in a preclinical osteosarcoma model [189]. An ongoing phase I/II study of second-generation HER2–CAR-T cells conducted in 19 patients with HER2-positive sarcomas showed two CRs and some disease stabilizations with evidence of tumor necrosis in patients with osteosarcoma without DLTs [182]. Another member of the ErbB family of receptor tyrosine kinases, EGFR (or HER1), is also being tested in a phase I trial of second-generation EGFR-CAR-T cells in children and young adults with EGFR-positive solid tumors. Preliminary results in the first 11 patients treated so far demonstrated mixed responses on day 28 after administration and tolerated additional CAR-T infusion without dose-limiting toxicity [190].

Among other target antigens that were developed and tested in preclinical models, glypican 3 (GPC3) and B7H3 are being tested in clinical trials on pediatric solid tumors (Table 3). Two phase I trials are examining the safety of B7H3–CAR-T in children and young adults with B7H3-positive R/R solid tumors. For one of these studies, preliminary results are available, showing three stabilizations of disease with a partial metabolic response and without DLTs in the nine subjects treated so far [191].

The efforts to find the best targets for immune cells directed to the treatment of pediatric solid tumors have also been paralleled by studies aimed at increasing the persistence and expansion of cell products in vivo through the co-expression/secretion of homeostatic cytokines or TME-modulating factors or at conferring resistance to immunosuppressive molecules [169].

## 6. TME Modulation Using NK Cells or Macrophages

NK cells are effectors of innate immunity and enhancers of adaptive immune responses through cytokine secretion; in addition, they play a role in antitumor immunity alone or in combination with antibodies through antibody-dependent cell-mediated cytotoxicity. Thanks to their highly cytotoxic, non-MHC-restricted effector function, NK cells have high potential for development as immunotherapies against cancer and have, thus, been tested in clinical trials as alternative ATMPs—either unmanipulated or gene-modified [192]. In preclinical studies, Ewing sarcoma, rhabdomyosarcoma, and osteosarcoma cells were demonstrated to be sensitive to killing by NK cells [193]. Clinical trials have been conducted in hematologic and solid tumors, and they have mainly enrolled adult patients. The results of early trials showed good tolerability but limited efficacy; subsequent trials endeavored to increase efficacy by coupling NK-based ATMPs with ICIs or other agents, but the results are not yet available [194]. NK-cell-based CAR therapies represent a promising alternative to the limitations of CAR-T, since clinical-grade off-the-shelf products can be generated from multiple allogeneic sources with a favorable safety profile and low risk of GvHD, neurotoxicity, and CRS. Most clinical trials have been conducted for hematologic malignancies in adult cohorts [194], although studies of solid tumors are ongoing [192].

TAMs have a tumor-promoting role within the TME; thus, strategies for counteracting their effects are being investigated [195]. In addition to blocking the recruitment of TAMs or depleting them [196], macrophages can be re-polarized to increase tumor cell phagocytosis or exhibit an inflammatory phenotype. An alternative to TAM alteration/reprogramming is the hypothesis of *educating* macrophages through in vitro culture and employing them in vivo to repopulate the TME and alter the pro-tumor environment. Early trials of polarized myeloid-based ATMP had limited efficacy but did not show dose-limiting toxicities [195]. More recently, macrophage engineering has been proposed as a tool to obtain more potent antitumor activity. CAR–macrophages targeting HER2 have been demonstrated to reduce tumor growth in animal models [197,198] and are now being tested in a phase I study for HER2-expressing solid tumors in adults alone or in association with pembrolizumab (NCT04660929).

## 7. Combined Approaches

The clinical benefit of ICIs or ATMPs alone in pediatric solid tumors has generally been limited, which is likely due to the peculiar features of the TME, including the scarcity of TILs, which outbalances the immune network in favor of an immunosuppressive environment. Providing more effective tumor-specific T cells while modulating the immunosuppressive fractions of the TME with ICIs or with other repolarizing interventions may be a key factor in obtaining better clinical results in pediatric solid tumors.

For this reason, an effort to combine different agents to increase efficacy while reducing toxicity is ongoing. The administration of ICIs in patients with lymphomas that are refractory or relapsing after CD19–CAR-T cells has been shown to provide clinical benefit and revert the exhausted profile of circulating CAR and non-CAR T-cells [199]. Studies addressing a combined approach in pediatric solid tumors are not yet available but could provide a needed breakthrough.

The synergistic effect observed with combined CAR-T cells and ICIs has led to the development of armored CAR-T cells secreting checkpoint inhibitors. The secretion of PD-1 single-chain variable fragment (scFv) by PD-1-expressing CAR-T cells enhanced T cell proliferation and reduced PD-1 expression in vitro. Additionally, anti-PD-1 secretion was seen to enhance CAR-T cell antitumor function in a mouse model of gastric cancer [200]. Similar effects of anti-PD-1 scFv secretion by CAR T-cells were reported in another study, which showed increased T cell cytokine production and proliferation in vitro, while in vivo, there was enhanced antitumor efficacy and CAR-T cell accumulation in the TME [201]. Several clinical trials with ICI-expressing CAR-T cells are currently recruiting adult patients and will hopefully also be available for pediatric patients.

Efforts are needed not only to combine ICIs and ATMPs but also to find the best schedule for delivering conventional therapies and novel biologicals. Toward this aim, multicenter efforts are critical in designing clinical trials in the pediatric setting.

## 8. Conclusions

Immunotherapeutics are changing the outcomes of adult hematologic and solid tumors. Although they have been successfully employed in pediatric hematologic cancer, their use in pediatric solid tumors has met with limited success, and for this reason, excluding a few exceptions, immunotherapy has been reserved to resistant/relapsed cases so far. The challenges met so far are due to the peculiar characteristics of the pediatric tumor microenvironment, the limited knowledge of the TME in childhood cancer due to the rarity of many pediatric tumors, the lack of reliable biomarkers and clinical assays for informing treatment decisions, and the difficulties in conducting clinical trials in a pediatric setting. One such challenge is antigen escape due to the loss or mutation of the target antigen, which could be addressed through the use of pharmaceutical strategies that upregulate the expression of cell-surface target antigens on cancer cells or through the use of dual-target strategies. Furthermore, poor T cell trafficking and persistence contribute to the lack of success of immunotherapies against solid tumors. The therapeutic strategy that is most likely to succeed in pediatric patients is a multimodal approach that combines chemotherapy and targeted therapies with the use of cell therapy to recruit adaptive tumor-specific T cells and/or NK cells to the TME while modulating their function and neutralizing the role of suppressor cells through ICIs and other agents. Immunoprofiling may help in the identification of immune signatures that are predictive of response, which are currently lacking in children.

A deeper understanding of the TME can help expand therapeutic options, uncover causes of resistance, and favor the development of innovative functional models for testing therapeutic agents [202,203,204]. Moreover, insight into the composition of the TME in pediatric cancer may suggest more specific approaches to the modulation of the immunosuppressive tumor environment and provide a rational basis for different associations of biological therapies, as well as their integration with conventional anti-cancer treatments, to achieve a greater impact on the outcomes of pediatric solid tumors.

## Figures and Tables

**Table 2:** Published and ongoing clinical trials of ICIs registered on clinicaltrials.gov as of January 2023 for pediatric solid tumors.

Target	Compound	Study Phase	Disease	Clinical Results/ Toxicity	Reference/ NCT#
CTLA-4	Ipilimumab	Phase I	Advanced ST	4 SD/33 pts enrolled; 27% grade 3–4 toxicities	[147]
PD-L1	Atezolizumab	Phase I/II	Pediatric ST and HD or NHL	4 PR/87 pts enrolled; 1 PR in 75 ST Good tolerability	[148]
PD-1	Pembrolizumab	Phase I/II	Pediatric ST and HD or NHL	2 CR, 15 PR/155 pts enrolled; 8 PR in ST 8% grade 3–5 toxicities	[149]
PD-1	Nivolumab	Phase I/II	Children/AYA ST HD or NHL	No objective responses in ST; 33% SD in sarcomas and 50% SD in neuroblastoma 36% grade 3–4 toxicities	[150]
PD1 + CTLA-4	Nivolumab + Ipilimumab	Phase II	R/R Pediatric ST	2 PR in young adults; 4 SD 6 DLTs/55 eligible pts	[151]
PD1 + CTLA-4	Nivolumab vs. Nivolumab + Ipilimumab	Phase I/II	High-grade pediatric CNS ST	No advantage of combined treatment Acceptable toxicity	[151]
PD-1	Nivolumab	Phase I/II (active, not recruiting)	Pediatric patients with hypermutated ST	2 patients with adenoCa and astrocytoma with prolonged PR	02992964 [152]
PD-1	Nivolumab + Metronomic CY	Phase I/II	Pediatric patients with R/R ST	2 unconfirmed PR acceptable toxicity, mostly hematological	[152]
PD-L1	Avelumab	Phase I	Pediatric patients with R/R ST	4 SD/21 pts enrolled; no grade 4–5 toxicities	[152]
PD-L1	Avelumab	Phase I	Pediatric patients with R/P osteosarcoma	n.a.	03006848
PD-1	Nivolumab + Dinutuximab + 131-mIBG	Phase I	Pediatric patients with neuroblastoma	n.a.	02914405
PD-1	Nivolumab	Phase I/II	Pediatric and adult patients receiving allo-HSCT for Sarcomas	n.a.	03465592
PD-1	Nivolumab + chemotherapy	Phase I/II	Pediatric patients with R/R ST	n.a.	03585465
PD-1	Nivolumab	Phase I/II	R/R Pediatric ST	n.a.	02901145
PD1 + CTLA-4	Nivolumab + Ipilimumab+ cryoablation	Phase II	R/R Pediatric ST	n.a.	05302921
PD-1/ PD-L1	Anti PD-1 or PD-L1 + levantinib	Phase II/III	Children with hepatoblastoma	n.a.	05322187

#: number; ST: solid tumor; SD: stable disease; HD: Hodgkin’s disease; NHL: non-Hodgkin lymphoma; PR: partial response; AYA: adolescents and young adults; R/R: refractory/relapsed; DLT: dose-limiting toxicity; CNS: central nervous system; CY: cyclophosphamide; allo-HSCT: allogeneic hematopoietic stem cell transplantation; R/P: recurrent/progressive.

**Table 3:** Published (ref.) or ongoing (NCT number) clinical trials registered on clinicaltrials.gov as of January 2023 for representative CAR-T cell therapies in pediatric solid tumors.

Target	Patient Nr.	Study Phase	Disease	Clinical Results/ Toxicity	Reference/ NCT#
GD2	11	Phase I	R/R neuroblastoma	3/11 CR no DLT	[180,181]
GD2	11	Phase I	R/R neuroblastoma	no objective responses, 4 SD (2 CR after additional treatment) no DLT	[155]
GD2	12	Phase I	R/R neuroblastoma	no objective responses, 3 SD no DLT	[180,181]
GD2	27	Phase I/II	R/R neuroblastoma	9 CR, 8 PR (36% 3-y EFS) 74% CRS (94% grade 1–2); hematologic toxicities	[182]
GD2 *	3 **	Phase I	R/R neuroblastoma	1 PR; no DLT; hematologic toxicities	03294954 [180,181]
GD2	4 **	Phase I	Children and adults with H3K27M-mutated gliomas	3/4 pts had radiological/clinical benefit Mild CRS; no on-target, off-tumor toxicity	04196413 [182]
GD2 *	n.a.	Phase I	Children and adults with R/R neuroblastoma or osteosarcoma	n.a.	03721068
HER2	19 **	Phase I/II	Children or AYA with R/R HER2+ sarcomas	Study ongoing; 2 CR (ongoing), 3 SD no DLT	00902044 [182]
GPC3	n.a.	Phase I	Children or AYA with R/R GPC3-positive ST	n.a. currently enrolling liver tumors	02932956
GPC3 *	n.a.	Phase I	Children or AYA with R/R GPC3-positive ST	n.a.	04377932
GPC3 ***	n.a.	Phase I	Children or AYA with R/R GPC3-positive ST	n.a.	04715191
EGFR	11 **	Phase I	Children or AYA with R/R EGFR-positive ST	3 mixed responses; no DLT	03618381 [163]
B7H3	9 **	Phase I	Children or AYA with R/R ST	no objective responses; 3 SD no DLT	04483778 [164]
B7H3	n.a.	Phase I	Children or AYA with R/R ST	n.a.	04897321

#: number; * IL-15 expressing CAR-T; ** study ongoing; *** IL-15/IL-21 expressing CAR-T. GD2: disialoganglioside GD2; R/R: refractory/relapsed; CR: complete response; SD: stable disease; DLT: dose-limiting toxicity; PR: partial response; CRS: cytokine release syndrome; HER2: human epidermal growth factor receptor 2; AYA: adolescents and young adults; GPC3: glypican 3; ST: solid tumors; EGFR: epidermal growth factor receptor; B7H3: B7 homolog 3.

## Abbreviations

**Acronym**	**Definition**	**Acronym**	**Definition**
APC	Antigen-presenting cell	NY-ESO-1	New York esophageal squamous cell carcinoma 1
ATMP	Advanced-therapy medicinal product	PD-1	Programmed cell death protein 1
CAR-T	Chimeric antigen receptor–T cell	PD-L1	Programmed death ligand 1
CSC	Cancer stem cell	scFv	Single-chain variable fragment
CTLA-4	Cytotoxic T-lymphocyte-associated protein 4	TAM	Tumor-associated macrophage
CTLs	Cytotoxic T lymphocytes	TCR	T cell receptor
ECM	Extracellular matrix	TFH	T follicular helper
EGF	Epidermal growth factor	TGF	Transforming growth factor
EGFR	Epidermal growth factor receptor	TIGIT	T-cell immunoreceptor and ITIM domains
HER2	Human epidermal growth factor receptor 2	TIL	Tumor-infiltrating lymphocyte
ICI	Immune checkpoint inhibitor	Tim-3	T cell immunoglobulin and mucin domain 3
IDO	Indolamine-2,3-dioxygenase	TMB	Tumor mutational burden
LAG-3	Lymphocyte activation gene-3	TME	Tumor microenvironment
MDSC	Myeloid-derived suppressor cell	TNF	Tumor necrosis factor
MMP	Matrix metalloprotease	Treg	Regulatory T cell
MYCN	N-myc proto-oncogene	VEGF	Vascular endothelial growth factor
